# A multi-center distributed learning approach for Parkinson's disease classification using the traveling model paradigm

**DOI:** 10.3389/frai.2024.1301997

**Published:** 2024-02-07

**Authors:** Raissa Souza, Emma A. M. Stanley, Milton Camacho, Richard Camicioli, Oury Monchi, Zahinoor Ismail, Matthias Wilms, Nils D. Forkert

**Affiliations:** ^1^Department of Radiology, Cumming School of Medicine, University of Calgary, Calgary, AB, Canada; ^2^Hotchkiss Brain Institute, University of Calgary, Calgary, AB, Canada; ^3^Biomedical Engineering Graduate Program, University of Calgary, Calgary, AB, Canada; ^4^Alberta Children's Hospital Research Institute, University of Calgary, Calgary, AB, Canada; ^5^Department of Medicine (Neurology), Neuroscience and Mental Health Institute, University of Alberta, Edmonton, AB, Canada; ^6^Department of Radiology, Radio-oncology and Nuclear Medicine, Université de Montréal, Montréal, QC, Canada; ^7^Centre de Recherche, Institut Universitaire de Gériatrie de Montréal, Montréal, QC, Canada; ^8^Department of Clinical Neurosciences, Cumming School of Medicine, University of Calgary, Calgary, AB, Canada; ^9^Department of Psychiatry, University of Calgary, Calgary, AB, Canada; ^10^Clinical and Biomedical Sciences, Faculty of Health and Life Sciences, University of Exeter, Exeter, United Kingdom; ^11^Department of Pediatrics, University of Calgary, Calgary, AB, Canada; ^12^Department of Community Health Sciences, University of Calgary, Calgary, AB, Canada

**Keywords:** traveling model, federated learning, distributed learning, Parkinson's disease, multi-center

## Abstract

Distributed learning is a promising alternative to central learning for machine learning (ML) model training, overcoming data-sharing problems in healthcare. Previous studies exploring federated learning (FL) or the traveling model (TM) setup for medical image-based disease classification often relied on large databases with a limited number of centers or simulated artificial centers, raising doubts about real-world applicability. This study develops and evaluates a convolution neural network (CNN) for Parkinson's disease classification using data acquired by 83 diverse real centers around the world, mostly contributing small training samples. Our approach specifically makes use of the TM setup, which has proven effective in scenarios with limited data availability but has never been used for image-based disease classification. Our findings reveal that TM is effective for training CNN models, even in complex real-world scenarios with variable data distributions. After sufficient training cycles, the TM-trained CNN matches or slightly surpasses the performance of the centrally trained counterpart (AUROC of 83% vs. 80%). Our study highlights, for the first time, the effectiveness of TM in 3D medical image classification, especially in scenarios with limited training samples and heterogeneous distributed data. These insights are relevant for situations where ML models are supposed to be trained using data from small or remote medical centers, and rare diseases with sparse cases. The simplicity of this approach enables a broad application to many deep learning tasks, enhancing its clinical utility across various contexts and medical facilities.

## 1 Introduction

Distributed learning, and especially its federated learning (FL) implementation, has emerged as a viable and promising alternative to central learning for training of machine learning (ML) models to address various patient privacy regulations and administrative barriers (Tuladhar et al., 2022). It provides a practical solution for accessing extensive and diverse datasets by facilitating ML model training in distributed environments. In the standard FL setup, each center receives a copy of a global model from a central server and local training takes place at each center for a pre-defined number of epochs using the data locally available. After local training, the learned model parameters are sent back to the server. Using an aggregation function, the server combines these parameters to update the global model, which is then sent back to the centers for additional training and model refinement. This iterative process is usually repeated over multiple rounds to improve the global model's performance (McMahan et al., 2016).

Prior studies that explored this standard FL setup for disease classification tasks based on medical images often employed large databases with a limited number of participating centers where the data were acquired, and/or used such data to generate artificial centers to simulate diverse data contributions. Artificial centers with imbalanced contributions are typically generated using the Dirichlet distribution, resulting in an overall distribution that exhibits exponential decay. This means that many centers will contribute many datasets (i.e., many medical images) while a few centers will contribute a small number of datasets. For instance, Yan et al. (2021) used a COVID-19 database consisting of 15,282 chest X-ray images and five artificially generated centers. Cetinkaya et al. (2021) employed a COVID-19 database containing 28,833 chest X-ray images and 20 artificial centers based on the Dirichlet distribution. Liu et al. (2023) evaluated their FL approach for COVID-19 (5,908 chest X-rays) and skin lesion detection (10,015 images) tasks by artificially creating centers (8, 9, 10, 11, 12 centers for COVID-19 and 3, 4, 5, 6, 7 centers for skin lesion detection) using the Dirichlet distribution. Additionally, Wicaksana et al. (2023) developed a FL model for skin lesion and intracranial hemorrhage classification using 23,247 dermatoscopy images and 67,969 brain CT images, respectively, with six centers for skin lesion detection and two artificial centers for intracranial hemorrhage classification. Jiang et al. (2022) developed and evaluated a FL model for breast cancer classification using 450,000 histology images from five centers, while Adnan et al. (2022) simulated data distributions across 4, 8, 16, and 32 centers for histopathology classification using 30,070 images. Li et al. (2020) evaluated their FL approach using 370 resting-state fMRI data from four centers. Lastly, Zhou et al. (2022) created 20 artificial centers for diabetic retinopathy classification using a FL system trained with a total of 3,662 images.

Although all the studies mentioned above offered important technical advancements and insights, their scope was inherently limited by the number of (real) centers participating in the distributed learning setup. Moreover, even though artificial centers with dissimilar contributions were simulated by sampling from a Dirichlet distribution, most of the centers still contributed a large number of datasets. However, such conditions may not effectively represent real-world scenarios for 3D imaging data where some centers may only have access to a very few datasets and the disease of interest may present differently across centers, raising concerns about the performance of FL with genuinely diverse and skewed data distributions that arise from the limited data available at medical facilities (Ng et al., 2021; Tuladhar et al., 2022).

The traveling model (TM) paradigm, also known as Cyclical Weight Transfer (CWT) (Chang et al., 2018; Balachandar et al., 2020), is an alternative approach to the standard FL setup for distributed learning. In an initial analysis by Souza et al. (2022b), TM has been shown to outperform FL for cases where limited datasets are available at each participating center. Although their examination is based on empirical evidence, the results suggest that TM holds promise as a potential alternative to FL in these particular scenarios. Briefly described, in the TM setup, a single model undergoes sequential training across various centers following a predetermined travel sequence that dictates the order of center visits. The model is initialized at a central server or the first center and undergoes training with the available data at that center. Subsequently, the updated model travels to the next center, where it continues training with the locally available data. This process continues until the final center is reached, completing one training cycle. Similar to FL, multiple cycles can be performed to improve the global model's performance. However, unlike in the standard FL setup, there is no need for an aggregation function as the same model is continuously improved by traveling from center to center.

The benefit of the TM for small local datasets stems from the iterative training of a single model, addressing the challenge of local models yielding suboptimal parameters due to overfitting, which often occurs when training ML models with very small sample sizes. Furthermore, this approach overcomes the challenge of aggregating multiple models without marginalizing centers with fewer datasets. However, in contrast to FL, the TM paradigm has seen limited exploration so far, with only one study specifically focusing on small sample sizes available in each center (Souza et al., 2022b). Moreover, the TM has not been used and evaluated for training convolution neural networks (CNNs) for disease classification using real distributed 3D imaging data where some centers provide only very few training samples. Instead, similar to FL studies, TM investigations (Chang et al., 2018; Balachandar et al., 2020; Souza et al., 2022b) often make use of large databases with simulated centers, prompting concerns regarding their genuine applicability to real-world scenarios.

Therefore, this work aims to develop and evaluate a Parkinson's disease (PD) classifier utilizing the TM approach. This traveling model classifier is developed and evaluated using a large database comprising 1,817 three-dimensional T1-weighted brain magnetic resonance imaging (MRI) scans acquired in 83 different real centers around the world. Each of these centers contributes distinct and unique information, encompassing biological (e.g., sex, age, and target labels) and non-biological (e.g., scanner types and the number of participants per center) factors. Our major contributions include: (1) the development and evaluation of a TM approach for training 3D CNNs for a disease classification purpose using 3D datasets, and (2) the first work to make use of a real-world data distribution with many centers providing only very few training samples, acquired using a wide selection of MRI scanners and acquisition protocols.

## 2 Materials and methods

In this study, we developed and trained a CNN model for PD classification from T1-weighted brain MRI data using a TM approach and analyzed its performance using the largest multi-center PD database described in the literature.

### 2.1 Dataset

All analyses conducted in this study utilized a distinct multi-center PD database, comprising 1,817 T1-weighted MRI scans acquired in 83 different healthcare centers around the world^1^, ^2^, ^3^ (Acharya et al., 2007; Jack et al., 2008; Hanganu et al., 2014; Sudlow et al., 2015; Badea et al., 2017; Wei et al., 2018; Duchesne et al., 2019; LaMontagne et al., 2019; Lang et al., 2019; Talai et al., 2021; Thibeau-Sutre et al., 2022). Each center received ethics approval from their local ethics board and received written informed consent from all the participants under the declaration of Helsinki. This database is exceptionally diverse, encompassing variations in participant demographics, center contributions, scanner vendors (Siemens, GE, and Phillips), scanner types (23 scanners were utilized), and magnetic field strengths (1.5T or 3.0T). Table 1 provides an overview of the database demographics.

**Table 1:** Database demographics.

		**Parkinson's disease**		**Healthy participants**			
**Centers or study**	Number of centers*	**Sex (M/F)**	**Age (<60/60+)**	**Sex (M/F)**	**Age (<60/60+)**	**Total amount of data**	**Scanner vendor** ^*^
ADNI	42	–	–	96/177	18/255	564	Siemens, GE, and Philips
BIOCOG	1	25/20	0/45	28/21	0/49	94	Siemens
C-BIG	1	36/30	16/50	1/9	3/7	76	Siemens
HAMBURG	1	52/22	23/51	24/15	13/26	113	Siemens
CCNA	6	37/20	8/47	–	–	57	GE
JAPAN	1	13/17	4/26	7/8	4/11	45	Siemens
NEUROCON	1	16/10	4 /22	4/12	6/10	42	Siemens
OASIS	1	–	–	17/10	5/22	27	Siemens
PD MCI CALGARY	1	53/26	0/79	20/22	0/42	121	GE
PD MCI PLS	1	26/15	15/26	10 /11	7/14	62	Siemens
PPMI	24	228/136	147/237	108/58	132/217	530	Siemens, GE, and Philips
SALD	1	–	–	78/0	34/44	78	Siemens
TAOWU	1	8/9	1/16	12/8	3/17	37	Siemens
UKBB	1	28/20	4/44	119/78	37/160	245	Siemens

^*^Per center and scanner type detailed information can be found in Supplementary Table 1.

Whenever possible, datasets from each center were divided into 80% for training and 20% for testing, resulting in 1,410 MRI scans for training and 407 MRI scans for testing. For centers providing a very small number of samples (e.g., <25 samples), the data were split into training and testing sets aiming to achieve an overall balanced representation in terms of sex and age, as shown in Table 2. All datasets were pre-processed as described in Souza et al. (2023), which included skull-stripping, resampling to an isotropic resolution of 1 mm, bias field correction, affine image registration to the PD25-T1-MPRAGE-1mm brain atlas (Xiao et al., 2017), and cropping to reduce irrelevant background information.

**Table 2:** Database split distribution.

**Participant characteristics**	**Total (train/test)**	**Number of males (train/test)**	**Number of females (train/test)**
Parkinson's disease	680/187	418/124	262/63
Healthy participants	730/220	400/120	330/100

### 2.2 Parkinson's disease model

**Figure 1:**
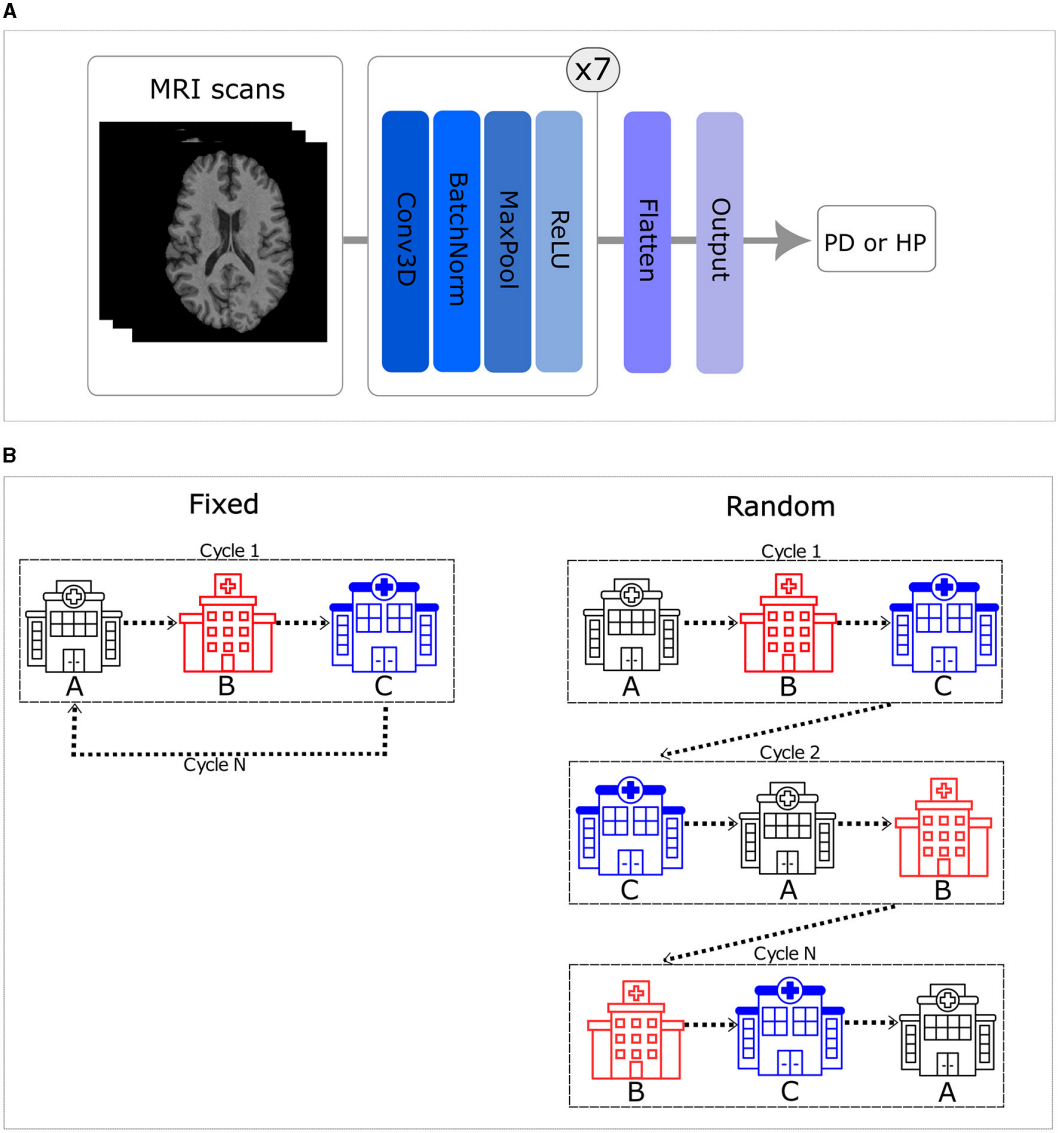
**(A)** Simple fully convolutional neural network (CNN) architecture. **(B)** Order of the training set. The left side illustrates that when trained in a fixed order, all centers are visited in a consistent sequence. In contrast, the right side shows that the sequence varies for each cycle when trained in a random order.

In this study, we utilized a state-of-the-art simple fully convolutional network (SFCN) (see Figure 1A), which achieved a high, state-of-the-art accuracy (78.8%) differentiating healthy participants and patients with PD using multi-center T1-weighted MRI scans in a centralized approach (Camacho et al., 2023), as the basis for all experiments. A grid search was conducted to optimize this centralized model on the data available for this work, considering various parameters such as learning rate, dropout layer, and learning rate decay. The best model was selected based on early stopping criteria, with a patience of 10 epochs, considering the lowest testing loss. The chosen model utilized the Adam optimizer with an initial learning rate of 0.001 and employed an exponential decay rate every epoch. The training was performed using a shuffled batch size of 5 and a dropout layer before the flattening layer, with a dropout rate of 20%.

### 2.3 Traveling model pipeline

In this study, we implemented the first CNN model for PD classification from T1-weighted brain MRI data using a TM approach. Moreover, we explored six distinct configurations of the CNN trained using the TM approach. These configurations encompassed random and fixed traveling sequences, with variations in the number of local training epochs (one, two, and five) prior to moving to the next center. The fixed traveling order involves visiting every center in a consistent sequence throughout each cycle, which is defined, using a random seed equal to 42, once in the beginning. In contrast to that, the random order introduces cycle-to-cycle variability, using a different seed (i.e., adding 1 to the initial seed of 42 after each cycle), in the sequence visited by the model, practically emulating the batch shuffling process used in the centralized approach (see Figure 1B).

The performance of the traveling models was assessed for up to 30 cycles, ensuring consistency with the total number of epochs used to train the centralized model, where each epoch corresponds to a full cycle in the traveling setup. With respect to the optimizer and dropout layer, the only difference between the traveling models and the centralized model is the initial learning rate, which was set to 0.0001. This value was chosen after conducting a grid search and taking into account that several centers have fewer than five samples (4, 3, 2, or 1) available for local training, necessitating a smaller learning step. As a result, the traveling model experiments utilized a batch size of 5 or equal to the number of samples available at a local center, if this number was smaller than 5. Each sample in the batch corresponds to a unique participant T1-weighted MRI scan. Although the training was conducted on a single computer equipped with an NVIDIA GeForce RTX 3090 GPU, the training procedure adhered to the TM concept by fetching data from a single center at any given time. Nevertheless, the outcomes outlined in this study are expected to remain consistent and unaffected by the specific physical implementation (such as computer network and data transfer protocols) as long as each center employs the identical hardware and software configuration utilized in our training. Our code is available at https://github.com/RaissaSouza/pd-travelling-model.

### 2.4 Evaluation metrics

For quantitative evaluation of our results, we measured the Area Under the Receiver Operating Characteristic Curve (AUC ROC), which provides a single scalar value that measures the overall threshold-independent performance of a binary classification model. More precisely, the AUC ROC score measures the model's capability to distinguish between positive (PD) and negative (healthy participants) classes across all possible thresholds. A higher AUC ROC score indicates a better predictive performance.

## 3 Results

**Figure 2:**
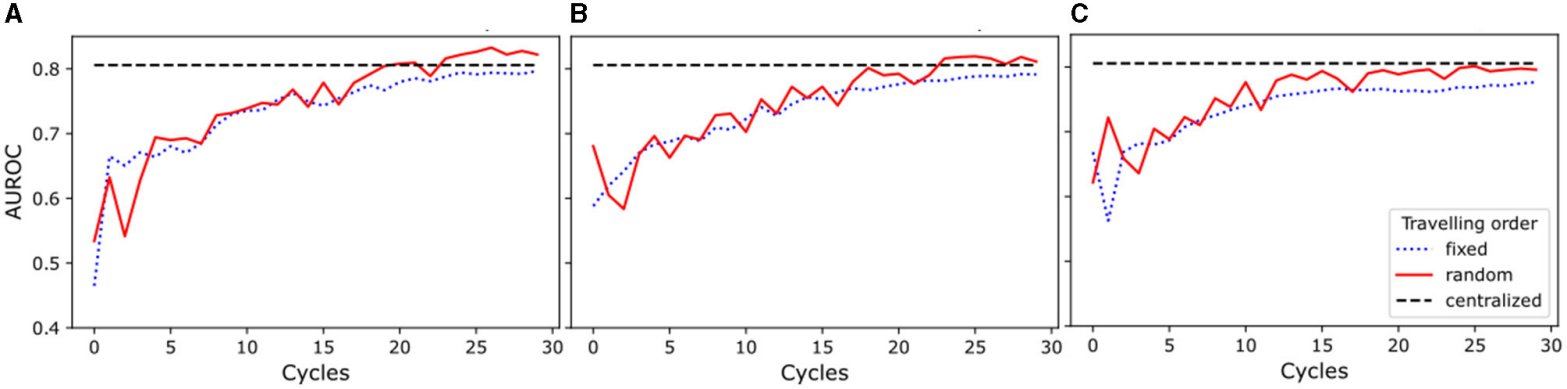
Area Under the Receiver Operating Characteristic Curve (AUROC) for experiments with random and fixed traveling order. The blue dotted line represents the results for the fixed traveling order, the red solid line represents the results for the random traveling order, and the black dashed line represents the best result for the centralized model AUROC after complete training. **(A)** Models trained for one local epoch. **(B)** Models trained for two local epochs. **(C)** Models trained for five local epochs.

The results of this study show (see Figure 2) that the random traveling order consistently outperformed the fixed traveling order across all experimental setups. As seen in Figures 2A, B, models trained for one and two local epochs employing the random traveling order achieved AUC ROC results comparable to the centralized model (80.57%) when trained for 24 cycles or more. In contrast, Figure 2C reveals that models trained for five local epochs exhibited inferior performance compared to the centralized model, regardless of the traveling order (detailed metrics per cycle and training scheme are presented in Supplementary Table 2).

Our results demonstrate that increasing the number of cycles improves the performance of the models for every setup investigated. Furthermore, our findings highlight that an increase in the number of local training epochs leads to greater instability (i.e., AUC ROC numbers vary between cycles) in the training process for the random traveling order, while conversely, it results in a smoother (i.e., less variability between cycles) trend for the fixed traveling order. Moreover, the model trained for a single local epoch and random traveling order displayed a more stable learning process when compared to the models that employed two and five local epochs and random traveling order. Additionally, Figure 2A shows a comparable level of stability in the training process for models trained with both fixed and random traveling orders for a single epoch.

## 4 Discussion

The main finding of this work is that the traveling model is suitable and leads to good results when used for training CNN models for disease classification using 3D imaging data distributed across many medical centers and limited data availability at single centers. Most notably, when trained for an appropriate number of cycles, the model trained in this distributed way achieves comparable or slightly superior performance compared to a standard model that was trained in centralized fashion. Moreover, the effectiveness of the traveling CNN model extends beyond scenarios where centers contribute limited samples, as previously shown in Souza et al. (2022b) using tabulated data. It also performs exceptionally well in this real-world data context characterized by diverse forms of imaging data distribution imbalances, including variations in target labels, scanner types, and demographics, as evident in this unique and realistic PD database. Importantly, our findings affirm the practical applicability of the traveling model paradigm for image-based classification systems in real-world contexts, enhancing its reliability for clinical deployment across multiple medical facilities.

Our findings reveal that employing a random traveling order and a single local epoch constitutes the optimal configuration for PD classification using this particular database. This setup exhibits greater stability in performance as a function of cycles compared to scenarios involving two or five local epochs, suggesting that an excessive amount of local training could potentially lead to model overfitting to the data provided by individual centers. Souza et al. (2022b) reported similar findings in their analysis of the effects of local training per cycle. However, it is important to note that their study differed from ours as they utilized simulated artificial centers, identical data distribution, and tabular data, while we used image data acquired in 83 real-world centers with non-identical data distribution, and full 3D images instead of tabulated data. Similar outcomes were also demonstrated in Souza et al. (2022a,c), where brain age prediction and brain tumor segmentation were examined. Nevertheless, noteworthy distinctions should be emphasized. In one instance, contributions from centers were simulated using tabular data, while in the other case, a CNN for segmentation was utilized, which has access to numerous positive and negative cases (voxels) within a single image. As a result, this study provides novel and highly relevant contributions to the field, showcasing the suitability of the traveling models approach for image-based disease classification tasks using data acquired in 83 real-world centers.

The observed smoother performance trend of the fixed traveling order could be attributed to several factors. One potential explanation is that the model might memorize the sequence of centers and exploit it as a shortcut. Another possibility is that the model consistently encounters the same center toward the end of each cycle leading to overfitting. In contrast, using a random order helps mitigate (at least partially) the memorization of the center order or the last center's local samples by emulating the batch shuffling process employed in centralized training. Another perspective is that this smoother performance trend might relate to the phenomenon of catastrophic forgetting (French, 1999; Kirkpatrick et al., 2017), where the model loses knowledge of previously learned patterns from initial centers and becomes overly specialized toward later-center data. Lastly, the smoothness could imply that the model's weight updates become minimal in later cycles, leading to diminishing improvements. On the other hand, the increased instability seen in the random traveling order suggests more pronounced weight updates across cycles, likely contributing to the observed performance variability.

In essence, our study highlights the efficacy of the traveling model for 3D medical image classification applications, particularly when dealing with limited training samples. These insights carry important implications for scenarios in which small centers or remote medical facilities are meant to contribute data, cases involving rare diseases (Taruscio et al., 2018) with limited case numbers even at major centers, and situations where centers predominantly serve pediatric patients, with considerable developmental differences (Rahimzadeh et al., 2018). The simplicity of our approach makes it versatile and applicable to a wide array of deep learning tasks and databases, thereby enhancing its clinical utility across diverse contexts.

It is essential to highlight some of the limitations of this work. First, our work made exclusively use of a single established PD classifier model. Thus, it remains to be shown that the results hold true if different deep learning models or disease models are considered. Nevertheless, it is worth highlighting that the multi-center database utilized in this study is notably extensive and encompasses a considerably larger number of centers compared to datasets employed in numerous other federated learning and traveling model analyses thus far, which makes it likely that the results are generalizable. Second, our study solely employed T1-weighted MRI sequences, thereby leaving out the exploration of alternative image modalities. Third, this work only simulated the network for the traveling model pipeline using the multi-center database. Therefore, future work investigating how to create such a distributed computer network in practice and how to define transfer protocols to send the model to different locations to train in distinct computers is necessary. Nevertheless, the results presented in this work should hold true if every center trains the model using the same hard- and software that we used. Lastly, the establishment of a metric to investigate catastrophic forgetting is necessary to determine the underlying cause of the varying stability observed in our model's training process.

## 5 Conclusion

This work explored and systematically investigated the applicability of the traveling CNN model paradigm for distributed training of a PD classifier using data acquired in 83 real centers around the world, exhibiting considerable heterogeneity in the data distribution per center, with the majority of centers contributing only a limited number of imaging samples. To the best of our knowledge, this is the first work making use of a large database of 3D images from real centers with limited local data to train an image-based disease classifier in a distributed way. Moreover, this is the first description of a novel distributed learning approach, specifically designed and evaluated for PD classification. Our results demonstrated that the traveling CNN model can achieve results similar to central learning. Thus, the traveling model provides a new opportunity to apply machine learning models to diverse and skewed data distributions as a result of limited data availability at medical facilities.

## Data availability statement

The original contributions presented in the study are included in the article/Supplementary material, further inquiries can be directed to the corresponding author.

## Ethics statement

Each center received ethics approval from their local ethics board and received written informed consent from all the participants under the declaration of Helsinki. The studies were conducted in accordance with the local legislation and institutional requirements. Written informed consent for participation was not required from the participants or the participants' legal guardians/next of kin in accordance with the national legislation and institutional requirements.

## Author contributions

RS: Conceptualization, Formal analysis, Funding acquisition, Investigation, Methodology, Visualization, Writing—original draft, Writing—review & editing. ES: Formal analysis, Writing—review & editing. MC: Data curation, Writing—review & editing. RC: Data curation, Writing—review & editing. OM: Data curation, Writing—review & editing. ZI: Data curation, Writing—review & editing. MW: Conceptualization, Formal analysis, Funding acquisition, Writing—review & editing. NF: Conceptualization, Formal analysis, Funding acquisition, Writing—review & editing.

## Footnotes

^1^ https://www.ppmi-info.org/

^2^ https://www.mcgill.ca/neuro/open-science

^3^ https://openneuro.org/datasets/ds000245/versions/00001
