# Supplementary material for: A multi-center distributed learning approach for Parkinson's disease classification using the traveling model paradigm
Source: Front Artif Intell. 2024 Feb 7;7:1301997. doi: 10.3389/frai.2024.1301997 (PMC10879577; doi:10.3389/frai.2024.1301997)
Supplement: Supplementary file 1 [file Data_Sheet_1.PDF]

Supplementary Material

Table S1: Demographics information per center

Disease status	Parkinson's disease		Healthy participants		Scanner type
Sites	Sex (M / F)	Age (<60 / 60+)	Sex (M / F)	Age (<60 / 60+)	
ADNI_1	-	-	3 / 3	0 / 6	Siemens Prima Fit
ADNI_2	-	-	8 / 14	1 / 21	Siemens Prisma and GE Signa Hdxt
ADNI_3	-	-	0 / 2	0 / 2	GE Discovery 750
ADNI_4	-	-	2 / 3	0 / 5	Philips Ingenia
ADNI_5	-	-	2 / 1	0 / 3	GE Signa Hdxt
ADNI_6	-	-	2 / 2	0 / 4	GE Discovery 750
ADNI_8	-	-	3 / 3	1 / 5	Siemens Prima Fit
ADNI_10	-	-	0 / 2	1 / 1	Siemens Biograph mMR
ADNI_11	-	-	3 / 9	3 / 9	Siemens Verio
ADNI_12			5 / 7	1 / 11	Siemens Skyra and GE Signa Hdxt
ADNI_13	-	-	0 / 2	0 / 2	Philips Achieva
ADNI_15	-	-	3 / 3	0 / 6	Siemens Prisma and GE Discovery 750
ADNI_16	-	-	6 / 3	1 / 8	GE Discovery 750 and GE Signa UHD
ADNI_19	-	-	0 / 2	0 / 2	Siemens Prima Fit
ADNI_20	-	-	7 / 1	0 / 8	GE Discovery 750 and GE Signa Hdxt
ADNI_21	-	-	1 / 1	0 / 2	GE Discovery 750 and Philips Achieva
ADNI_23	-	-	2 / 2	0 / 4	Siemens Prisma Fit
ADNI_24	-	-	2 / 4	0 / 6	Siemens Skyra
ADNI_26	-	-	0 / 5	0 / 5	Siemens Skyra and Siemens Prisma
ADNI_27	-	-	0 / 4	0 / 4	Siemens Prisma
ADNI_28	-	-	2 / 5	0 / 7	Siemens Prisma Fit
ADNI_33	-	-	0 / 2	0 / 2	Siemens Prisma Fit
ADNI_34	-	-	1 / 6	3 / 4	Siemens Trio Tim
ADNI_37	-	-	2 / 4	0 / 6	GE Signa Hdxt
ADNI_38	-	-	2 / 6	1 / 7	GE Discovery 750 and Siemens Prisma
ADNI_39	-	-	1 / 1	0 / 2	GE Signa Premier

Table S1: Demographics information per center

Disease status	Parkinson's disease		Healthy participants		Scanner type
Sites	Sex (M / F)	Age (<60 / 60+)	Sex (M / F)	Age (<60 / 60+)	
ADNI_40	-	-	2 / 3	1 / 4	GE Discovery 750 and Philips Achieva
ADNI_41	-	-	3 / 2	0 / 5	Philips Achieva
ADNI_42	-	-	1 / 2	0 / 3	Siemens Trio Tim
ADNI_43	-	-	4 / 5	0 / 9	Siemens Verio, Siemens Skyra, and Siemens Trio Tim
ADNI_44	-	-	3 / 6	0 / 9	Siemens Trio Tim
ADNI_47	-	-	4 / 8	0 / 12	GE Discovery 750
ADNI_49	-	-	2 / 8	1 / 9	GE Discovery 750
ADNI_50	-	-	2 / 6	0 / 8	Philips Achieva dStream
ADNI_51	-	-	0 / 2	1 / 1	Philips Ingenia
ADNI_52	-	-	5 / 2	0 / 7	GE Discovery 750
ADNI_54	-	-	1 / 1	0 / 2	Siemens Skyra
ADNI_55	-	-	3 / 9	0 / 12	Siemens Skyra and Siemens Verio
ADNI_58	-	-	6 / 16	1 / 21	Siemens Prisma Fit
ADNI_59	-	-	0 / 3	0 / 3	Siemens Prisma Fit and Philips Achieva
ADNI_60	-	-	3 / 5	2 / 6	GE Discovery 750 and Philips Ingenia
ADNI_61	-	-	0 / 2	0 / 2	Siemens Trio Tim and Philips Ingenia
BIOCOG	25 / 20	0 / 45	28 / 21	0 / 49	Siemens Sonata
C-BIG	36 / 30	16 / 50	1 / 9	3 / 7	Siemens Prisma Fit
HAMBURG	52 / 22	23 / 51	24 / 15	13 / 26	Siemens Skyra
HMC	1 / 2	0 / 3	-	-	GE Discovery 750
Japan_dataset	13 / 17	4 / 26	7 / 8	4 / 11	Siemens Verio
JGH	2 / 0	0 / 2	-	-	Siemens Trio Tim
MUC	7 / 3	3 / 7	-	-	Siemens Trio Tim and Siemens Prisma Fit
Neurocon	16 / 10	4 /22	4 / 12	6 / 10	Siemens Avanto
OASIS	-	-	17 / 10	5 / 22	Siemens Trio Tim and Siemens Biograph mMR
PD_MCI_CALGARY	53 / 26	0 / 79	20 / 22	0 / 42	GE Discovery 750
PD_MCI_PLS	26 / 15	15 / 26	10 /11	7 / 14	Siemens Trio Tim
PPMI_10	9 / 7	6 / 10	4 / 3	3 / 4	GE Discovery 750 and GE Signa Hdxt

Table S1: Demographics information per center

Disease status	Parkinson's disease		Healthy participants		Scanner type
Sites	Sex (M / F)	Age (<60 / 60+)	Sex (M / F)	Age (<60 / 60+)	
PPMI_12	10 / 9	5 / 14	5 / 5	4 / 6	Philips Achieva
PPMI_13	17 / 5	10 / 12	2 / 3	0 / 5	Siemens Trio Tim
PPMI_14	2 / 0	1 / 1	-	-	Siemens Trio Tim
PPMI_15	11 / 5	6 / 10	5 / 5	4 / 6	GE Optima MR450 and GE Signa Hdxt
PPMI_16	12 / 7	8 / 11	4 / 4	4 / 4	GE Signa Hdxt and Philips Gyroscan NT
PPMI_17	8 / 3	5 /6	6 / 3	5 / 4	GE Signa Hdxt
PPMI_18	7 / 6	2 / 11	4 / 0	2 / 2	Siemens Trio Tim
PPMI_19	15 / 7	11 / 11	8 / 4	9 / 3	Siemens Trio Tim and Siemens Espree
PPMI_20	20 / 17	22 / 35	6 / 7	7 / 6	GE Genesis Signa, GE Signa Excite, Siemens Espree, and Siemens Symphony
PPMI_21	11 / 3	9 / 5	-	-	Philips Gyroscan NT and Philips Intera
PPMI_22	14 / 4	5 / 13	10 / 2	3 / 9	Philips Achieva and GE Signa Hdxt
PPMI_23	3 / 9	6 / 6	9 / 3	4 / 8	Siemens Trio Tim and Siemens Espree
PPMI_25	14 / 5	7 / 12	7 / 2	5 / 4	Siemens Trio Tim
PPMI_26	8 / 6	5 / 9	1 / 0	0 / 1	GE Genesis Signa, GE Signa Hdxt, and Siemens Espree
PPMI_27	12 / 9	8 / 13	8 / 3	6 / 5	Siemens Trio Tim and GE Signa Hdxt
PPMI_28	13 / 7	9 / 11	4 / 1	2 / 3	Siemens Trio Tim
PPMI_29	4 / 7	2 / 9	6 / 0	3 / 3	Siemens Trio Tim, Siemens Espree, and Siemens Symphony
PPMI_30	2 / 1	1 / 2	2 / 0	0 / 2	Siemens Verio
PPMI_51	11 / 7	5 / 13	5 / 2	4 / 3	Siemens Trio Tim
PPMI_52	15 / 8	8 / 15	4 / 7	3 / 8	Siemens Trio Tim
PPMI_53	2 / 3	3 / 2	3 / 4	6 / 1	Siemens Verio
PPMI_55	3 / 0	1 / 2	1 / 0	0 / 1	Siemens Verio
PPMI_59	5 / 1	2 / 4	-	-	Philips Intera
RUH	3 / 3	0 / 6	-	-	Siemens Skyra

Table S1: Demographics information per center

Disease status	Parkinson's disease		Healthy participants		Scanner type
Sites	Sex (M / F)	Age (<60 / 60+)	Sex (M / F)	Age (<60 / 60+)	
SALD	-	-	78 / 0	34 / 44	Siemens Trio
SBK	3 / 0	0 / 3	-	-	Siemens Prisma
Taowu	8 / 9	1 / 16	12 / 8	3 / 17	Siemens Trio
UKBB	28 / 20	4 / 44	119 / 78	37 / 160	Siemens Skyra
UOA	21 / 12	5 / 28	-	-	Siemens Prisma

Table S2: AUC ROC per cycle and training scheme

Number of cycles	Models trained for one local epoch		Models trained for two local epochs		Models trained for five local epochs	
	Fixed travelling order	Random travelling order	Fixed travelling order	Random travelling order	Fixed travelling order	Random travelling order
1	0.46	0.53	0.59	0.68	0.67	0.62
2	0.66	0.63	0.62	0.61	0.56	0.72
3	0.65	0.54	0.64	0.58	0.67	0.66
4	0.67	0.63	0.67	0.67	0.68	0.64
5	0.66	0.69	0.68	0.70	0.68	0.70
6	0.68	0.69	0.69	0.66	0.69	0.69
7	0.67	0.69	0.69	0.70	0.71	0.72
8	0.69	0.68	0.69	0.69	0.72	0.71
9	0.71	0.73	0.71	0.73	0.72	0.75
10	0.73	0.73	0.71	0.73	0.73	0.74
11	0.74	0.74	0.72	0.70	0.74	0.78
12	0.74	0.75	0.74	0.75	0.75	0.73
13	0.75	0.74	0.73	0.73	0.75	0.78
14	0.76	0.77	0.74	0.77	0.76	0.79
15	0.75	0.74	0.76	0.75	0.76	0.78
16	0.74	0.78	0.75	0.77	0.76	0.79
17	0.75	0.75	0.76	0.74	0.77	0.78
18	0.76	0.78	0.77	0.78	0.76	0.76
19	0.77	0.79	0.77	0.80	0.76	0.79
20	0.77	0.80	0.77	0.79	0.77	0.79
21	0.78	0.81	0.78	0.79	0.76	0.79
22	0.79	0.81	0.78	0.78	0.76	0.79
23	0.78	0.79	0.78	0.79	0.76	0.80
24	0.79	0.82	0.78	0.82	0.76	0.78

Table S2: AUC ROC per cycle and training scheme

Number of cycles	Models trained for one local epoch		Models trained for two local epochs		Models trained for five local epochs	
	Fixed travelling order	Random travelling order	Fixed travelling order	Random travelling order	Fixed travelling order	Random travelling order
25	0.79	0.82	0.78	0.82	0.77	0.80
26	0.79	0.83	0.79	0.82	0.77	0.80
27	0.79	0.83	0.79	0.82	0.77	0.79
28	0.79	0.82	0.79	0.80	0.77	0.80
29	0.79	0.83	0.79	0.82	0.77	0.80
30	0.79	0.82	0.79	0.81	0.78	0.80
